# Incorporating PET information in radiation therapy planning

**DOI:** 10.2349/biij.3.1.e4

**Published:** 2007-01-01

**Authors:** M MacManus, T Leong

**Affiliations:** Department of Radiation Oncology, Peter MacCallum Cancer Centre, East Melbourne, Victoria, Australia

**Keywords:** PET, radiation therapy, treatment planning, dosimetry, cancer

## Abstract

PET scanning, because of its impressive sensitivity and accuracy, is being incorporated into the standard staging workup for many cancers. These include lung cancer, lymphomas, head and neck cancers, and oesophageal cancers. PET often provides incremental information about the patient’s disease status, adding to the data obtained from structural imaging methods, such as, CT scan or MRI. PET commonly upstages patients into more advanced disease categories. Incorporation of PET information into the radiotherapy planning process has the potential to reduce the risks of geographic miss and can help minimise unnecessary irradiation of normal tissues. The best means of incorporating PET information into radiotherapy planning is uncertain, and considerable effort is being expended in this area of research.

## INTRODUCTION

To plan and deliver “radical” (or potentially curative) radiotherapy accurately for patients with malignant tumours, the locations of the tumour and the tumour-bearing lymph nodes in three-dimensional space must be known. In addition, the relation of tumour to the anatomy of critical adjacent normal tissues must be considered. Conventional three-dimensional imaging modalities, such as, CT or MRI scanning have serious limitations in determining the true extent of tumour in many clinical situations. The advent of PET scanning has revolutionised our approach to many cancers, and it is beginning to have a major impact on radiotherapy planning at those centres where there is good access to this new imaging technology. Rapid changes have also been occurring in the technology of radiotherapy in recent years [1]. Highly advanced treatment planning systems now enable much more accurate and rapid three-dimensional radiation dose calculations. These permit quantitative volumetric assessment of dose distribution both in tumour and critical normal tissues [2]. Ionising radiation can now be delivered accurately to complex three dimensional shapes using linear accelerators with independently-controlled multileaf collimators. The beam of radiation can be dynamically shaped during the course of treatment delivery [3]. Highly flexible techniques, such as, Intensity-Modulated Radiotherapy (IMRT) are available [4], making it possible to shape the high dose volumes in complex ways. Therefore, the radiation dose may be escalated without increased toxicity [5], or the existing local control rates may be maintained with reduced toxicity [6].

CT scanning is the primary imaging modality for three-dimensional radiotherapy planning. It not only provides important three-dimensional anatomic information, but also forms the basis for radiation dosimetry by reconstruction of a three dimensional electron-density map. This map is crucial for calculating radiation absorption and scatter. Unfortunately, the information that CT scanning can provide about the location of the tumour is often insufficient by itself to accurately guide treatment. When tumours have similar imaging characteristics to surrounding normal tissues, as is often the case for lesions in the oesophagus, liver, spleen, or salivary glands, they may be completely invisible on CT. Geographic miss and ultimate treatment failure may be inevitable, or excessive dose may be delivered to regions that do not contain tumour. Accurate lymph node staging is crucial for treating moderately advanced cancers with curative intent [7, 8]. CT is often a poor investigation technique for lymph node staging, because nodal size alone is the criterion for assessing tumour involvement. Sensitivity for detection of small nodes containing tumour is very low, and false positives commonly occur due to benign reactive lymphadenopathy [9].

Staging with PET, primarily using ^18^F-fluorodeoxyglucose (FDG), has rapidly acquired a key role in the management of cancers, such as, non-small cell lung cancer (NSCLC) [10, 11], Hodgkin and non-Hodgkin lymphomas [12, 13], head and neck cancers, oesophageal cancers [14, 15], and cervical carcinomas [16, 17]. It is also effective for imaging malignant melanoma [18, 19], soft tissue sarcoma [20], gastrointestinal cancers [21], and some other less common cancer types [22, 23]. Information from PET can improve the results of radical radiation therapy simply by better selection of patients. Significantly improved survival has already been demonstrated in patients with NSCLC. They were selected for radical RT using PET, compared with a conventionally staged control group [24], largely because patients with PET-detected metastases and advanced locoregional disease were denied futile aggressive therapy. Accurate imaging of tumour in three-dimensions using PET has the potential to improve the quality of radiotherapy planning, minimise the risk of geographic miss, and allow normal tissues that do not contain gross tumour to be spared from unnecessary toxicity [25]. As PET has become more widely used by radiation oncologists, the question has arisen “What is the best method for incorporating PET information into treatment planning?” In the following sections, some aspects of RT planning with PET will be considered and examples will be given of how PET can influence radiation oncology practice in common thoracic cancers.

## PET AND THE RADIOTHERAPY PLANNING PROCESS

Manufacturers of treatment planning systems have become aware of the need to accommodate the importation and display of PET information in radiotherapy planning systems. The most up to date systems allow integration of PET-CT information directly into the RT contouring workstation. PET and CT information can readily be combined to produce a Biological Target Volume (BTV) [26], incorporating all available structural and functional imaging. For lung cancer and several other malignancies, possibly including oesophageal cancer, the BTV represents the best target for high dose irradiation that we can currently define. When clinical PET first become available to oncologists, no means existed for incorporating PET information directly into the treatment planning process. Typically, PET and diagnostic CT images were simply displayed side by side on a light box and the Radiation Oncologist would visually incorporate the PET information when contouring the GTV. This method works quite well for anatomical structures that are well-demarcated on CT. However, it is not a good method for helping to delineate the boundaries of larger FDG-avid tumours that have interfaces with normal tissues where there is low CT contrast between tumour and normal tissue. To combine PET and CT data effectively in these cases, it is necessary to devise some method for displaying PET and CT information simultaneously within the treatment planning software.

### Co-registration of separately-acquired PET and CT images for treatment planning

At our own centre and at other institutions, in-house methods were developed for importing PET information into the radiotherapy treatment planning system. We developed a system that used fiducial markers, applied to the patient at separate CT and PET image acquisitions [27]. For both scans, patients were positioned identically by radiation therapists using lasers installed in both the CT and the PET suites. Phantom studies showed that the method was highly reproducible and could be utilised in clinical practice. DICOM PET information was imported into the radiotherapy planning system (Cadplan) and displayed side by side with the corresponding CT image, using software developed at our institution. This proved highly successful in practice, but with the installation of our first combined PET/CT scanner, it has since been entirely superseded at our institution. This methodology is still widely used as PET/CT scanners are less commonly used than stand-alone PET scanners, although the rate of growth of PET/CT availability is very high, especially in the US.

### Use of integrated PET/CT for treatment planning

The modern combined PET/CT scanner provides the best means for determining the BTV and for planning RT in those cancers that are well imaged by PET. Because PET and CT images are acquired on the same gantry, there is no need for repositioning the patient. This represents a major advance on all older methods. Planning systems, such as, Focalease and Pinnacle enable seamless transfer of PET/CT data into the contouring workspace and provide a wide range of options for display of fused PET and CT images. For treatment planning, the PET/CT scanner should be fitted with a rigid couch top and the imaging suite should be equipped with a laser positioning system, identical to that used for simulation and RT treatment. Patients should be scanned in the treatment position with immobilisation devices fitted and ideally under the supervision of a radiation therapist/therapy radiographer. Following the scan, the PET/CT data can be transmitted to the treatment planning system in DICOM format.

### Delineation of Biological Target Volumes

After the PET/CT data are imported into the planning computer, the radiation oncologist is faced with a series of new challenges. How do I define the gross tumour volume (GTV) using PET and CT? What nodes should I regard as positive? How do I define the edge of a tumour when it imperceptibly fades into normal tissues? How do I account for tumour movement? There has been little guidance from the literature on how best to use PET/CT information in contouring tumour and target volumes. Some problems are relatively easy to deal with; a lymph node that is negative for tumour by CT criteria, but is unequivocally involved on PET, can easily be incorporated into the target volume. For these decisions, the opinion of an experienced nuclear medicine physician should always be sought. Changes to the perceived status of the thoracic lymph node stations have the biggest influence on changing target volumes in the treatment of NSCLC. Similarly, enlarged nodes that are not metabolically active on PET may be omitted from the GTV if considered unlikely to contain tumour, and this is simple to accomplish.

The boundaries of some tumours can be very difficult to define, especially those that do not have clearly delineated margins on CT component of PET/CT [28]. Motion of the patient on the couch top, which should be minimal with appropriate positioning and immobilisation, and internal motion also contribute to the blurriness of PET images. Other factors that commonly cause difficulty include regions of low avidity in the tumour due to necrosis, the confounding effects of inflammation and infection that can give rise to intense uptake well within the range of standardised uptake value (SUV) seen in tumours, and poor contrast between tumours with a low SUV and adjacent normal structures. PET information is acquired over many respiratory and cardiac cycles and, therefore, an “average” position of the structures is imaged. In contrast, CT image is acquired virtually instantaneously and usually at a random phase of the respiratory cycle.

Because of the potential for inter-observer variation in using PET to determine target volumes for RT, several methods have evolved that attempt to make the results as uniform as possible. These are essentially either visual methods using the skill of the human observer or automated methods using a mathematical algorithm to contour the edge of the tumour in a reproducible and unbiased way. Worldwide, the most common approach to contouring is the visual one, using the skill of the observer, and this is the method of choice at our institution. At the Peter MacCallum Cancer Centre, RT planning PET/CT data are displayed using uniform window settings and colour settings. All available information is used in an “intelligent” process, including PET and CT data, biopsy reports, and the results of fluoroscopy to assess tumour movement. We draw upon our institutional experience of comparing individual PET/CT scan information with surgical findings in operable cases to assist our interpretation of the PET scan. We recognise the limits of the training and the experience of radiation oncologists in PET. Therefore, contouring of the GTV is only carried out following consultation with the nuclear medicine physician, who is asked to draw around the edge of the tumour on a hard copy of the PET/CT scan. Uniform training and detailed guidelines are used to minimise bias in this process, although the final interpretation of the scan is always reliant on the judgement of a PET physician. As an alternative to marking a hard copy, the PET Physician may be present in the radiotherapy planning area at the start of planning. Preliminary, so far unpublished, results of a reproducibility study at our centre suggest that this approach gives very similar results whether the GTV is contoured by a radiologist, PET physician, or Radiation Oncologist. The level of reproducibility is extremely high. This is in contrast to the poor results seen at other centres when CT alone is used for contouring, suggesting that PET/CT images are easier to interpret.

An alternative approach to contouring recognises that visual methods require a high level of human skill and experience and might exhibit significant variability between observers. This approach makes use of the quantitative information available from PET. Various automated or semi-automated approaches are under evaluation for tumour contouring [26, 29-31], but none of these methods has yet proven to be a trustworthy solution to the problem in vivo [32]. This method can produce good results with static phantoms [33] because FDG uptake is a result not only of malignant processes, but can arise due to a range of inflammatory and physiological processes. A simple FDG intensity map or a function derived from it cannot be a general solution for contouring. Some processes that can be readily recognised by an experienced physician (e.g., uptake in brown fat or muscle, sarcoidosis) have the potential to confound a purely quantitative approach. An increase in reproducibility could potentially lead to a decrease in accuracy unless each computer-derived tumour contour is edited by the Radiation Oncologist, correcting instances where normal or benign areas of uptake are wrongly interpreted as tumour by the software.

### Movement

Tumours in living subjects are not stationary, but move to varying degrees within the patient. This internal range of movement is referred to as the Internal Target Volume or ITV [31, 33]. The ITV is often well-appreciated on PET scanning because of the long acquisition time. Metabolically active lesions are usually qualitatively elongated in the axes of respiratory movement compared with their true dimensions, reflecting temporal blurring of activity, unless the CT image is acquired using some form of gating. Due to the lesion spending relatively less time at the extremes of respiratory excursion, particularly at the end of inspiration, activity tends to be less intense in those axial planes where the tumour spends proportionally less time. The PET-determined volume, therefore, indicates very clearly the region where a tumour spends maximum time. Consequently, a treatment based on the PET determined GTV is likely to be more accurate than a treatment based on a randomly acquired CT image [34] [35].

## SPECIFIC EXAMPLES OF THE ROLE OF PET IN RADIATION THERAPY PLANNING

### Non-Small Cell Lung Cancer

Lung cancer is the malignancy in which PET has had the greatest impact on selection of patients for radiotherapy and on radiotherapy planning [36]. This relates both to the clarity of imaging of a metabolically active cancer in a location favourable for PET and to the high rate of incremental abnormal findings seen on PET, compared with conventional imaging [37]. There is an abundance of evidence from surgical series, with systematic clinico-pathological correlation, proving that PET is much more accurate than CT in the assessment of thoracic lymph nodes, especially when CT and PET information are combined. Dwamena and colleagues reviewed English-language reports on the performance of PET (14 studies, 514 patients) and/or CT (29 studies, 2,226 patients) [38]. They reported that FDG-PET was significantly more accurate than CT (P < .001). Mean sensitivity was 0.79 for PET vs 0.60 for CT. Mean specificity was 0.91 PET vs 0.77 for CT. These data can be extrapolated to radiotherapy candidates, for whom mediastinoscopy is usually not performed if the patient clearly has unresectable disease on the basis of CT findings or is medically unfit for surgery. PET has had the greatest impact on RT planning in patients for whom PET shows different lymph node status from CT, most commonly upstaging the extent of apparent nodal disease. A high impact is also seen in patients with atelectasis, where the boundary between tumour and collapsed/consolidated lung can only be identified with the aid of PET [39].

A number have studies have tried to quantify the impact of PET on RT planning in NSCLC. In our own earliest study, we used PET rather that PET/CT and had no ability to co-register images [10]. Despite those limitations, we found that 22 out of 102 patients had a significant increase in RT target volumes to cover new sites of disease seen only on PET. In 16 patients, the target volume was reduced because regions of bland atelectasis could be excluded or enlarged nodes proved not to be FDG-avid. In 1998, Nestle and Colleagues reported that a significant increase in the radiation field was required to cover PET detected disease in 9% of 34 patients, but a significant decrease was seen in 26%, especially those with atelectasis [39]. Munley and colleages recorded that 35% of 35 patients had an increase in the RT field as a result of PET [40]. In a larger study of 73 patients, Vanuytzel and colleagues found that there was an increase in GTV in 22% of patients and a decrease in 40% [41]. Other significant studies include work by Bradley and colleagues who used co-registered sequential PET and CT scans and reported increased GTV in 46% and reduced GTV in 12% [42]. Brianzoni and colleagues reported that GTV/CTV was increased in 44% and reduced in 6% of 24 lung cancer patients planned using a dedicated PET/CT scanner [43].

### Oesophageal Cancer

Oesophageal cancers are usually locally and /or regionally advanced at presentation, and only a small proportion (20%) of patients can be cured by surgery alone. Combined chemoradiation with or without surgery is commonly used to treat this disease, and the use of concurrent chemoradiation has been found to significantly increase overall survival and cure rates compared with radiotherapy alone. As with lung cancer, CT is usually relied upon to determine the target volume for radiotherapy, and incorporation of PET data into RT planning has the potential to improve the accuracy of the process [44]. Although the radial extent of oesophageal tumours can usually be defined with CT, it is well recognised that the longitudinal extent is more difficult to define. This problem is compounded by the rising incidence of adenocarcinoma of the distal oesophagus and gastro-oesophageal junction where the distal extent of the tumour (often in the cardia of the stomach) can at times be impossible to visualise on CT. CT is also often inaccurate when used to estimate the extent of nodal involvement. PET is significantly more accurate than CT for the assessment of nodes [45] that are not immediately adjacent to the oesophagus and can more accurately delineate the longitudinal extent of tumour than CT. This is especially useful in cases where an endoscope is unable to pass through a stenosed oesophagus to visualise the lower boundary of the tumour.

**Figure 1:**
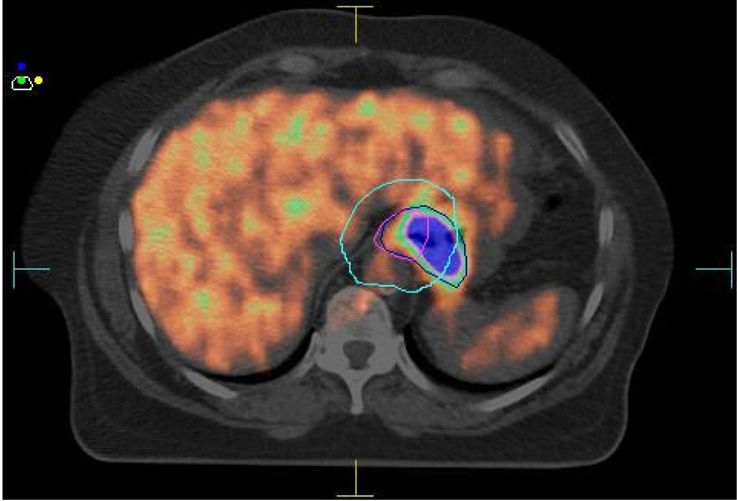
Treatment planning PET/CT scan in oesophageal carcinoma, illustrating differences between PTV determined by CT (pale blue) and PET/CT (dark blue). Some gross tumour lie outside the PTV determined using CT alone.

Our group has demonstrated that FDG PET has a significant impact on patient management in a multi-disciplinary setting both in selecting patients for neoadjuvant chemoradiation [46] and also following it, but prior to definitive surgery [47]. In both these settings, the findings on FDG PET were strongly predictive of survival, irrespective of management chosen. Results from a prospectively conducted trial of PET in RT planning, also conducted at our centre, show that PET has a significant impact on RT target volumes in oesophageal cancer [48]. In our cohort of patients the GTV based on CT data alone excluded PET-avid disease in 69% of patients. In 31% of patients this would have resulted in a geographic miss due to inadequate coverage of the primary oesophageal tumour and exclusion of unsuspected nodal disease from the irradiated volume. An example of a patient where CT and PET/CT target volumes differed significantly is shown in figure 1. In another study, Moureau-Zabotto and colleagues reported that the addition of PET information to CT-based RT planning altered the GTV in 19 of 34 patients (56%), with a reduction in the GTV in 12 patients (35%) and an increase in 7 (21%) [49]. These promising results suggest that routine incorporation of PET data into radiotherapy planning could improve the results of treatment for oesophageal carcinoma.

## CONCLUSION

Even the most sceptical radiation oncologist becomes an enthusiast for PET when this technology becomes available for RT treatment planning. The very high rate of incremental information and the increased accuracy associated with this powerful imaging modality are rapidly changing our approach to some of the most common cancers. It is essential that we work hard to find the best ways to incorporate this new information into our everyday practice.
